# Establishing and characterizing a new primary effusion lymphoma cell line harboring Kaposi’s sarcoma–associated herpesvirus

**DOI:** 10.1186/s13027-016-0086-5

**Published:** 2016-08-17

**Authors:** Madori Osawa, Sohtaro Mine, Shinichiro Ota, Kengo Kato, Tsuyoshi Sekizuka, Makoto Kuroda, Michiyo Kataoka, Hitomi Fukumoto, Yuko Sato, Takayuki Kanno, Hideki Hasegawa, Keiji Ueda, Masashi Fukayama, Takuya Maeda, Soichiro Kanoh, Akihiko Kawana, Yuji Fujikura, Harutaka Katano

**Affiliations:** 1 Department of Pathology, National Institute of Infectious Diseases, 1-23-1 Toyama, Shinjuku-ku, Tokyo, 162-8640 Japan; 2 Military Medicine Research Unit, Test and Evaluation Command, Japan Ground Self Defense Force, 1-2-24 Ikejiri, Setagaya, Tokyo, 154-0001 Japan; 3 Department of Pathology, Graduate School of Medicine, The University of Tokyo, Hongo 7-3-1, Tokyo, 113-0033 Japan; 4 Division of Infectious Diseases and Respiratory Medicine, Department of Internal Medicine, National Defense Medical College, 3-2 Namiki, Tokorozawa, Saitama 359-8513 Japan; 5 Pathogen Genomics Center, National Institute of Infectious Diseases, 1-23-1 Toyama, Shinjuku-ku, Tokyo, 162-8640 Japan; 6 Division of Virology, Department of Microbiology and Immunology, Osaka University Graduate School of Medicine, 2-2 Yamada-oka, Suita, Osaka 565-0871 Japan

**Keywords:** Primary effusion lymphoma, PEL, Kaposi’s sarcoma–associated herpesvirus, KSHV, Cell line

## Abstract

**Background:**

Primary effusion lymphoma is a rare distinct large B-cell neoplasm that is associated with Kaposi’s sarcoma–associated herpesvirus (KSHV) infection. Over recent years, 9 KSHV-positive/Epstein-Barr virus (EBV)-negative PEL cell lines have been established.

**Methods:**

Tumor cells were collected from the pleural effusion of a 49-year-old male with AIDS. Cells were grown in RPMI1640 culture medium supplemented with 10 % fetus bovine serum. Single cell cloning was performed successfully by a limiting dilution method in a 96-well plate. The cell line obtained was designated SPEL.

**Results:**

SPEL cells showed gourd-shaped morphology with a polarized nucleus, expressing CD38, CD138, and Blimp-1, but not B cell markers such as CD19 and CD20. Polymerase chain reaction analysis revealed that SPEL cells were positive for KSHV but negative for EBV. Tetradecanoylphorbol acetate induced expression of KSHV lytic proteins and the production of KSHV particles in SPEL cells. Subcutaneous inoculation of SPEL cells into severe combined immunodeficiency mice resulted in the formation of solid tumors. Next-generation sequencing revealed the 138 kbp genome sequence of KSHV in SPEL cells. Suberic bishydroxamate, a histone deacetylase inhibitor, induced the expression of KSHV-encoded lytic proteins and cell death in SPEL cells.

**Conclusions:**

A new KSHV-positive and EBV-negative PEL cell line, SPEL was established. This cell line may contribute to furthering our understanding of the pathogenesis of PEL and KSHV infection.

## Background

Kaposi’s sarcoma–associated herpesvirus (KSHV), or human herpesvirus 8 (HHV-8), is a member of gamma herpesvirus family, first isolated from Kaposi sarcoma lesions in AIDS patients [1, 2]. KSHV has since also been detected in certain lymphoproliferative disorders such as primary effusion lymphoma (PEL) and multicentric Castleman disease in AIDS patients [1]. PEL, also known as body-cavity-based lymphoma, is a rare distinct large B-cell neoplasm that accounts for about 4 % of all AIDS-related non-Hodgkin’s lymphomas [3, 4]. Patients with PEL are often also suffering from human immunodeficiency virus (HIV) infection or other immune deficiencies, such as solid organ transplant recipients [3, 5, 6]. PEL is typically diagnosed as a lymphomatous effusion in the pleural, pericardial, or peritoneal cavities, without forming solid tumors [7]. In 30–70 % of cases, Kaposi sarcoma is a complicating factor. KSHV is detected in the PEL cells without exception [7], whereas about 80 % of HIV-positive cases also present latent infection of Epstein–Barr virus (EBV) [8]. Histologically, PEL cells possess large, round to irregular-shaped nuclei with prominent nucleoli, and a variable amount of cytoplasm that is deeply basophilic and often vacuolated. PEL cells display a unique immunophenotype [3] expressing CD45 but not typical B-cell (including surface and cytoplasmic immunoglobulin, CD19, CD20, CD79a) and T-cell (CD3, CD4, CD8) markers. Instead, several markers of lymphocyte activation (CD30, CD38, CD70, human leukocyte antigen DR) and plasma cell differentiation (CD138) are usually detected [9, 10]. There is currently no standard treatment available for PEL. At present, a combination of highly active antiretroviral therapy with a CHOP (cyclophosphamide, doxorubicin, vincristine, prednisone)-like regimen is considered the first-line therapy [5, 6]. However, the prognosis of PEL is extremely poor with a median survival time of around 6 months and a 1-year survival rate of around 40 % [6].

Established cell lines are useful tools for investigating pathogenesis and treatment outcomes. Over recent years, 18 PEL cell lines have been established. Nine of these were co-infected with KSHV and EBV, while the other nine cell lines were EBV-negative [11–16]. In this report, we established a new KSHV-positive/EBV-negative PEL cell line, and demonstrated activation of KSHV and cell death in response to exposure to various drugs.

## Methods

### Patient

**Fig. 1:**
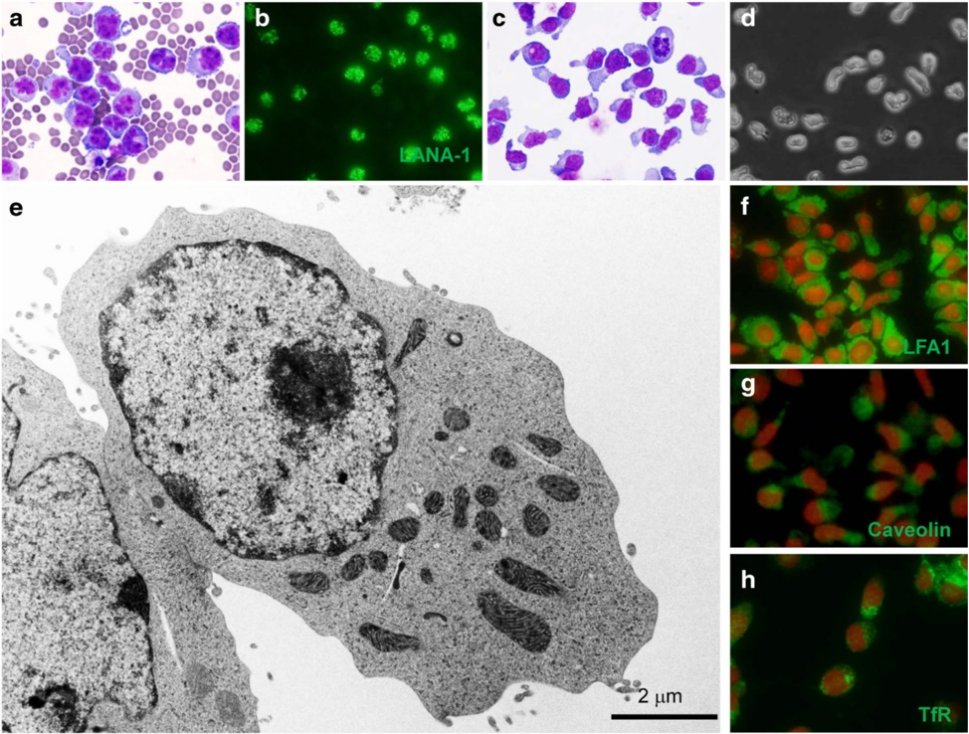
Morphology of SPEL cells and the corresponding original tumor cells. **a**, **b** Lymphoma cells in the pleural effusion of the patient. Giemsa staining (**a**) and an immunofluorescence assay for KSHV-encoded LANA-1 (**b**) are shown. **c** Giemsa staining of SPEL cells. **d** Phase-contrast image of SPEL cells in the culture medium. **e** Transmission electron microscopy of SPEL cells. **f**–**h** Immunofluorescence assay for LFA1 (BD Biosciences 610826, **f**), caveolin (Abcam ab2910, **g**), and transferrin receptor (TfR, 13-6800, Zymed Laboratories, South San Francisco, CA, USA, **h**) in SPEL cells

A 49-year-old Japanese male patient with AIDS was admitted to the hospital with a high-grade fever, extreme weight loss, and massive bilateral pleural effusion. His CD4 cell count was 31 cells/μL. Serologic tests showed that the patient was positive for hepatitis C and B virus antibody in the serum. Tumor cells in the pleural effusion were examined for Giemsa staining and flow cytometry, and finally diagnosed as CD20-, CD38+, CD138+, and KSHV-encoded latency-associated nuclear antigen 1 (LANA-1) + PEL (Fig. 1a and b).

### Establishing the cell line

The study protocol was approved by the Institutional Review Board, National Institute of Infectious Diseases (Approval No. 617) and the National Defense Medical College (Approval No. 2396). Tumor cells were collected from the pleural effusion and grown in RPMI1640 culture medium supplemented with 20 % fetus bovine serum (FBS). Supplementation with FBS was gradually reduced during passage. Finally, SPEL cells were grown in RPMI1640 culture medium supplemented with 10 % FBS. Single cell cloning was performed successfully by a limiting dilution method in a 96-well plate. The cell line obtained was designated SPEL.

### Flow cytometry

SPEL cells were fixed and permeabilized using BD Cytofix fixation buffer and BD Phosflow perm buffer III (BD Biosciences Pharmingen, San Diego, CA, USA). Cells were incubated for 1 h with mouse monoclonal antibodies, including CD3-ε, CD4, CD10, CD19, CD20, CD30, CD38, CD45, CD45-RO, CD79a, CD98, CD138, IgD, Ig κ, Ig λ, LFA1 (BD Biosciences Pharmingen), MDM2, VCAM (Santa Cruz Biotechnology, Santa Cruz, CA, USA), IgM (Life Technologies, Carlsbad, CA, USA), gp80 (Thermo Scientific, Waltham, MA, USA), and Blimp1 (Cell Signaling Technology, Danvers, MA, USA). The secondary antibody Alexa Fluor 488-conjugated anti-mouse IgG (Molecular Probes, Eugene, OR, USA) was then applied and the cells were incubated for 30 min. The cells were washed with stain buffer (BD Biosciences Pharmingen) three times and then analyzed using a flow cytometer (Cyflow counter, Partec, Görlitz, Germany). Data were analyzed using FlowJo software (Flow Jo, Ashland, OR, USA).

### Cytogenetic analysis

Chromosome slides were prepared using a short-term culture method. In brief, SPEL cells were suspended in a petri dish filled with the standard growth medium supplemented with 10 % FBS and 0.02 μg/mL colcemid. The cells were incubated for 20 min at 37 °C and harvested. Karyotype analysis was carried out at different passages choosing 20 well banded metaphases each time by Chromocenter (Tottori, Japan).

### Immunoglobulin gene rearrangement analysis

Immunoglobulin gene rearrangement analysis was performed by the BioMed2 method as described previously [17, 18].

### Polymerase chain reaction (PCR) analysis of virus genomes

KSHV and HIV DNA were amplified by conventional PCR [14]. EBV BNLF-1 DNA was amplified with the following primers: BNLF-1-forward (5ʹ–GCCAAAAGCTGCCAGATGGT-3ʹ) and BNLF-1-reverse (5ʹ-ACTGATGAGTAAGTATTACA-3ʹ). In addition, more than 160 virus genomes were examined with the multivirus real-time PCR system as described previously [19].

### Immunofluorescence assay

SPEL and BCBL-1 cells were cultured with or without tetradecanoylphorbol acetate (TPA) for 48 h. Suspended cells were then cytospun onto a glass slide. The samples were incubated with rabbit monoclonal antibodies to LANA-1, K8, vIL-6, or ORF59 diluted × 1000 with Block Ace, and mouse monoclonal antibody to replication and transcription activator (RTA) diluted × 500 with Block Ace for 1 h at room temperature, followed by incubation with Alexa Fluor 488-conjugated goat anti-rabbit (or anti-mouse) IgG (H + L) (Molecular Probes) diluted × 400 with PBS for 1 h at room temperature [20, 21]. Nuclear staining was performed with propidium iodide at 5 μg/ml. Imaging was performed using a fluorescence microscope (IX71, Olympus, Tokyo, Japan).

### Western blot analysis

Protein extraction and immunoblotting were performed as described previously [22]. Briefly, 1 × 10^6^ cells were lysed in 100 μL M-PER lysis buffer containing Halt protease and a phosphatase inhibitor cocktail (Pierce Biotechnology, Rockford, IL, USA). Cell lysates were subjected to sodium dodecyl sulfate polyacrylamide gel electrophoresis and transferred onto a polyvinylidene fluoride microporous membrane (Immobilon-P Transfer Membrane, Millipore, Bedford, MA, USA) using the NuPAGE system (Life Technologies). The membranes were blocked with Block Ace (DS Pharma Biomedical, Osaka, Japan) and probed with the antibodies to LANA-1 [23], RTA [21], vIL-6 [20], ORF59 [24], or Lyn (H-6, sc-7274, Santa Cruz Biotechnology), followed by a horseradish peroxidase-conjugated anti-mouse antibody (Promega, Madison, WI, USA) with an immunoreaction enhancer solution (Can Get Signal, Toyobo, Osaka, Japan). Blots were visualized using Super-Signal West Femto Chemiluminescent Substrate (Thermo Fisher Scientific).

### Northern blotting

SPEL and BCBL-1 cells were cultured with or without TPA for 48 h. Total RNA was extracted from cells using the Isogen RNA extraction kit (Nippon Gene, Tokyo, Japan). Then, 10-μg mRNA samples were separated on a 1.9 % formaldehyde-containing agarose gel, transferred to a nylon membrane (Roche Molecular Biochemicals, Indianapolis, IN, USA), and hybridized with K8 and β2-microglobulin probes. The probes were labeled by PCR with digoxigein-11-dUTP (Roche Molecular Biochemicals) [25].

### Quantitative real-time PCR

SPEL and BCBL-1 cells were cultured with or without TPA for 48 h. DNA and RNA were extracted from the cells using the DNeasy Blood & Tissue Kit and RNeasy Mini Kit (Qiagen GmbH, Hilden, Germany), respectively. The KSHV copy number was analyzed by real-time PCR for ORF26 DNA. The DNA copy numbers per cell were calculated by dividing the ORF26 copy numbers by half of the beta-actin copy numbers, because each cell had two copies of the gene in two alleles. KSHV mRNA transcripts were also detected by real-time reverse-transcription (RT)-PCR of RTA, which is expressed in the immediate early phase of KSHV infection. Primer and probe sequences for ORF26 and RTA have been reported previously [26, 27]. Real-time PCR or RT-PCR were performed on an Mx3005P PCR system (Agilent Technologies, Santa Clara, CA, USA).

### KSHV real-time PCR array

SPEL and BCBL-1 cells were cultured with or without TPA for 48 h. RNA samples were extracted using an RNeasy Mini Kit (Qiagen). To determine the expression profiles of KSHV gene transcripts, a KSHV real-time PCR array was performed using the TaqMan real-time RT-PCR system according to a previous report [22]. This system was designed to analyze all of the 87 KSHV gene transcripts simultaneously. Each value was normalized to the copy number of GAPDH transcripts, and the ratio of the values for treated (TPA for 48 h) versus untreated cells was calculated.

### Electron microscopy

TPA-treated SPEL cells were pelleted by centrifugation, fixed with 2.5 % glutaraldehyde and 2 % paraformaldehyde in 0.1 M phosphate buffer (pH 7.4) for 2 h at room temperature, post-fixed in 1 % osmium tetroxide and embedded in Epon. Ultrathin sections were stained with uranyl acetate and lead citrate, and observed under a transmission electron microscope (H7700, Hitachi High Technologies, Tokyo, Japan) at 80 kV.

### Whole-genome sequencing of KSHV using a next-generation sequencer

KSHV particles were collected from 75 mL of culture supernatant of 10 μM of suberic bishydroxamate (SBHA)-stimulated SPEL cells. The culture supernatant was subjected to ultracentrifugation at 100,000 × *g* for 2 h. The pellet was treated with DNase and RNase for 1 h. After heat inactivation at 70 °C for 10 min, viral DNA was extracted from the pellet. A DNA library was prepared with KAPA HyperPlus kit (Kapa Biosystems, Wilmington, MA, USA) with five cycles of PCR enrichment, followed by gel extraction for purification of the DNA library. Next-generation sequencing was performed using a MiSeq Reagent Kit v3 (600-cycle; Illumina, San Diego, CA, USA). Sequence reads were trimmed and assembled using the VirusTAP pipeline software (https://gph.niid.go.jp/cgi-bin/virustap/index.cgi) [28]. Sequence gap regions were amplified by conventional PCR, and sequenced using the Sanger method. The complete genome sequence of SPEL KSHV was deposited in the GenBank database under accession no. AP017458.

### Multiple sequence alignments and the KSHV genotype

Comparative KSHV full genome analysis was performed using the progressive alignment option available in the Mauve software (version 20150226, Darling Laboratory at the University of Technology Sydney). Four KSHV genomes, GK18 (GenBank accession no. AF148805.2), BCBL-1 (HQ404500.1), DG-1 (JQ619843.1), and JSC-1 (GQ994935.1), were compared with the SPEL KSHV sequence. Nucleotide sequences of the KSHV K1 region were aligned and a phylogenetic tree was constructed using the NJ-plot method and the Genetyx software (Genetyx, Tokyo, Japan). In addition to our samples, 17 previously reported K1 sequences were obtained from the GenBank database and used as reference sequences for comparison with the sequences in this study [29].

### Inoculation of the SPEL cell line into severe combined immunodeficiency mice

SPEL cells (5 × 10^7^) were subcutaneously injected into the neck of three 10-week-old CB17 severe combined immunodeficiency (SCID) mice [27]. All procedures were approved by the Animal Care and Use Committee of the National Institute of Infectious Diseases (NIID, Approval No 115123) and were conducted according to the ‘Guidelines for Animal Experiments Performed at the NIID’.

### Histology and immunohistochemistry

Histological analysis and immunohistochemistry for LANA-1 was performed as described previously [27].

### Drug screening

SPEL cells were cultured with eight histone deacetylase (HDAC) inhibitors (SBHA and HDAC inhibitor Set II: Sigma Aldrich, St Louis, MO, USA) and 26 other antitumor drugs (Table 1) for 48 h. Cells were stained with trypan blue and cell viability was measured using a TC10 automated cell counter (BioRad, New York, NY, USA). Simultaneously, RNA was extracted from the drug-stimulated cells, and RTA and GAPDH mRNA copies were measured by real-time RT-PCR [27].

**Table 1:** Drugs used for screening in SPEL cells

Drug	Company, Catalog no.	Category	Concentration
No drug	-	Control	-
DMSO (Dimethyl sulfoxide)	Sigma-Aldrich, 34869	Control	-
Doxorubicin	Kyowa-Kirin, Adriacin	Anti-cancer drug	10 μM
Vincristine	Nippon-Kayaku, Oncovin	Anti-cancer drug	10 μM
Etoposide	Sigma-Aldrich, E1383	Anti-cancer drug	10 μM
Anti-IgM SA-DA4	Abcam, ab99740	Anti-IgM	10 μM
Anti-IgM FC	Abcam, ab772	Anti-IgM	10 μM
Anti-IgM UNLB	Southern Biotech, 2020-01	Anti-IgM	10 μM
IC-261	Sigma-Aldrich, I0658	Casein kinase inhibitor	10 μM
TBB	Sigma-Aldrich, T0826	Casein kinase inhibitor	10 μM
D4476	Sigma-Aldrich, D1944	Casein kinase inhibitor	10 μM
TPA (tetradecanylphorbol acetate)	Sigma-Aldrich, P8139	Dicarboxylic acid	20 ng/mL
SBHA (suberic bishydroxamate)	R &D Systems, 38937-66-5	HDAC inhibitor	10 μM
Trichostatin A	Sigma-Aldrich, T8552	HDAC inhibitor	10 μM
Cl994	Sigma-Aldrich, C0621	HDAC inhibitor	10 μM
SAHA	Sigma-Aldrich, SML0061	HDAC inhibitor	10 μM
Tubacin	Sigma-Aldrich, SML0065	HDAC inhibitor	10 μM
Scriptaid	Sigma-Aldrich, S7817	HDAC inhibitor	10 μM
Panobinostat	Sigma-Aldrich, EPI009	HDAC inhibitor	10 μM
Sodium Butyrate	Sigma-Aldrich, B5887	HDAC inhibitor	1.25 μM
SC514	Santa Cruz, sc-205504	IKKb inhibitor	10 μM
Thalidomide	Sigma-Aldrich, T144	Immunomodulatory drug	10 μM
Pomalidomide	Sigma-Aldrich, P0018	Immunomodulatory drug	10 μM
Lenalidomide	Santa Cruz, sc-218656	Immunomodulatory drug	10 μM
Cyclophosphamide	Sigma-Aldrich, C7397	Immunosuppressant	10 μM
AG490 (Tyrphostin AG 490)	Sigma-Aldrich, T3434	JAK inhibitor	10 μM
SP600125	Sigma-Aldrich, S5567	JNK inhibitor	10 μM
Rapamycin	Sigma-Aldrich, R8781	mTOR inhibitor	10 μM
BAY11-7082	Sigma-Aldrich, B5556	NF-kB inhibitor	10 μM
LY-294002	Sigma-Aldrich, L9908	PI3K inhibitor	10 μM
Z-leu-leu-leu-H	Peptide Institute, 3175-v	Proteasome inhibitor	10 μM
Bortezomib	Calbiochem, 5.04314.0001	Proteasome inhibitor	10 μM
Simvastatin	MSD, Lipovas	Statin	10 μM
Lovastatin	Sigma-Aldrich, 1370600	Statin	10 μM
Prednisolone	Shionogi, Predonine	Steroid	10 μM
Dexamethasone	Sigma-Aldrich, D4902	Steroid	10 μM

## Results

### Establishment of the SPEL cell line and its morphological features

At first, the primary culture of the tumor cells from the pleural effusion grew in RPMI1640 culture medium supplemented with 20 % FBS. However, after a few passages, autonomous growth was observed in RPMI1640 culture medium with 10 % FBS. The 30th passage was accomplished on the 150th day of culturing. After single cell cloning, cells showed a gourd-shaped morphology with a polarized nucleus (Fig. 1). Single cloned cells were designated as SPEL (Saitama-PEL). Electron microscopy also demonstrated that the polarized cytoplasm in the gourd-shaped SPEL cells contained abundant mitochondria and Golgi apparatus (Fig. 1e). An immunofluorescence assay revealed that caveolin and transferrin receptor localized predominantly in the polarized cytoplasm, whereas LFA1 was expressed broadly (Fig. 1f–h).

### Immunological and genetic characteristics of SPEL cells

**Fig. 2:**
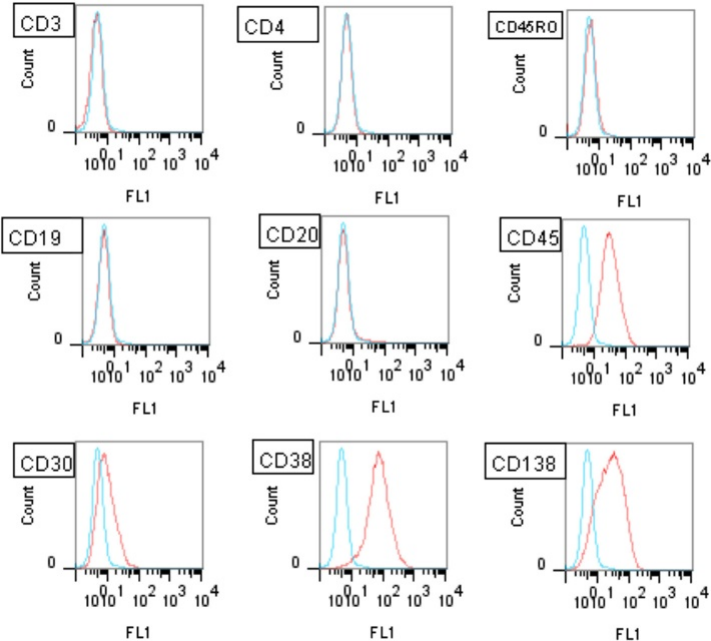
Flow cytometry of SPEL cells. Red and blue lines indicate specific and control antibodies, respectively

**Fig. 3:**
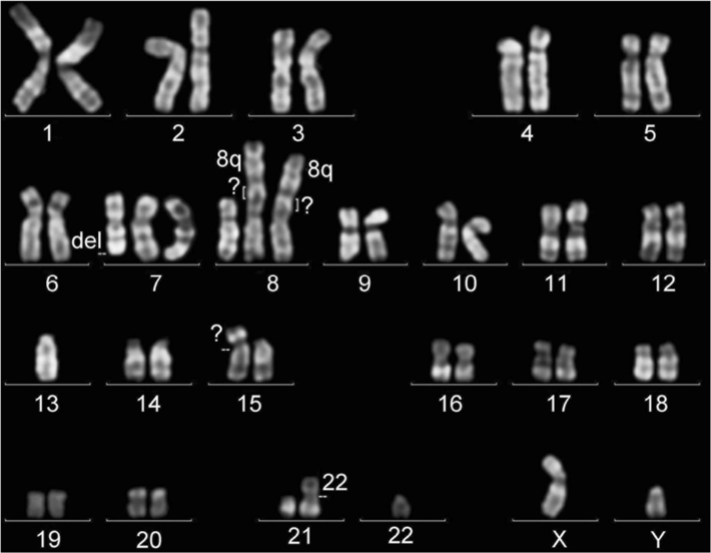
Chromosomal karyotype of SPEL cells. An image of the Q-band is shown

**Fig. 4:**
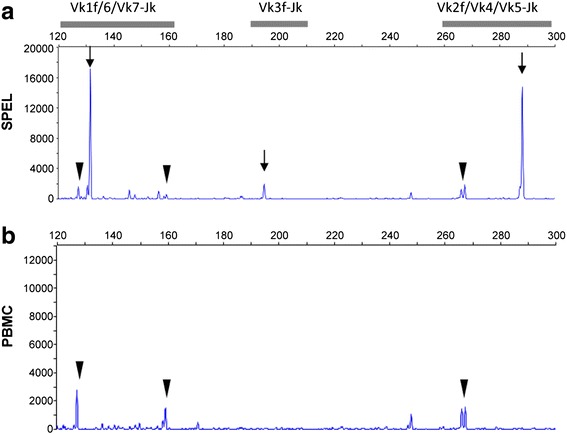
Genetic rearrangement of the immunoglobulin κ chain as analyzed by the BioMed2 method. **a** SPEL cell line. **b** Peripheral blood mononuclear cells (PBMC) from another unrelated patient. Fragment analysis of PCR products of the IGK tube A using the BioMed 2 method is shown. PCR products of Vk1f/6/Vk7-Jk, Vk3f-Jk, and Vk2f/Vk4/Vk5-Jk were observed in the 120–160, 190–210, and 260–300 bp regions, respectively. Arrows indicate clonal bands. Arrow heads indicate products of the germ bands in each region

**Fig. 5:**
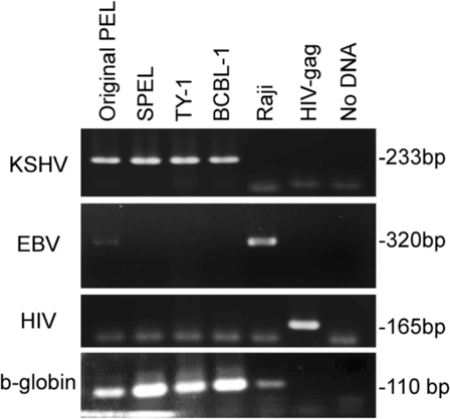
PCR analysis of virus infection in SPEL cells. KSHV ORF26, the EBV BNLF-1 region, and the HIV gag gene were amplified by conventional PCR. The human beta-globin gene was included as an internal control. TY1 and BCBL1 are KSHV-positive, EBV-negative, cell lines. Raji is an EBV-positive Burkitt lymphoma cell line. A plasmid containing the HIV-gag region was used as a HIV control

Flow cytometry analysis revealed that SPEL cells were positive for CD30, CD38, CD138, CD4, CD45 and Blimp-1, LFA1, MDM2, IL-6, VCAM, MyD88, gp80, IgD, IgM, IgG, and Ig light chain κ, and negative for CD3, CD10, CD19, CD20, CD45RO, CD79a, CD98, and Ig light chains λ (Fig. 2). These immunophenotypic features suggested that SPEL cells were derived from the plasma cell lineage. Chromosomal analysis revealed that the karyotype of SPEL cells was 46, XY, +del(7)(q32), +8, der(8)t(8;8)(p21;q13)ins(8;?)(p21;?) × 2, 13, add(15)(p11), der(21;22)(q10;q10)) (Fig. 3). BioMed2 analysis [17] revealed that SPEL cells had an immunoglobulin κ gene rearrangement, indicating the B cell lineage (Fig. 4). PCR analysis of the DNA of SPEL cells clearly indicated that they are a KSHV-positive, EBV-negative PEL cell line (Fig. 5). The multivirus real-time RT-PCR system [19] showed that SPEL cells were positive for KSHV but negative for more than 160 other viruses including EBV, HIV-1, hepatitis B virus, and hepatitis C virus (data not shown).

### Induction of KSHV lytic infection in SPEL cells by TPA

**Fig. 6:**
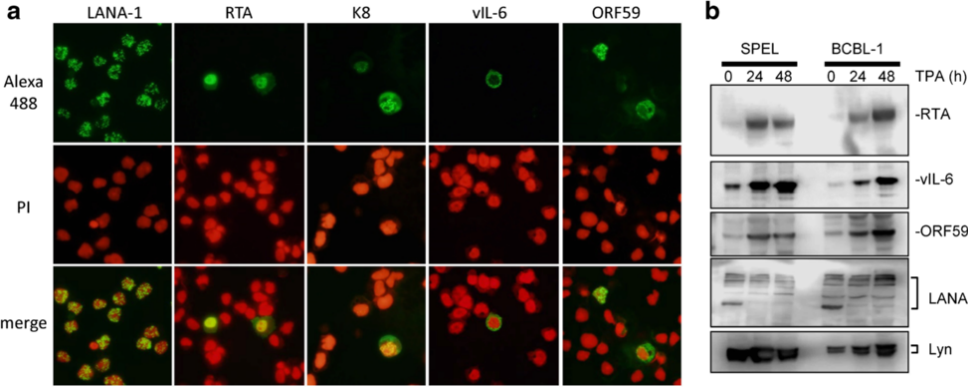
Expression of KSHV-encoded protein in TPA-stimulated SPEL cells. **a** Immunofluorescence assay for KSHV-encoded proteins. PI (propidium iodide) was used as a counter stain for the nucleus. **b** Western blot analysis. Lysates of 0, 24, and 48 h-TPA stimulated PEL cells were electrophoresed and blotted

**Fig. 7:**
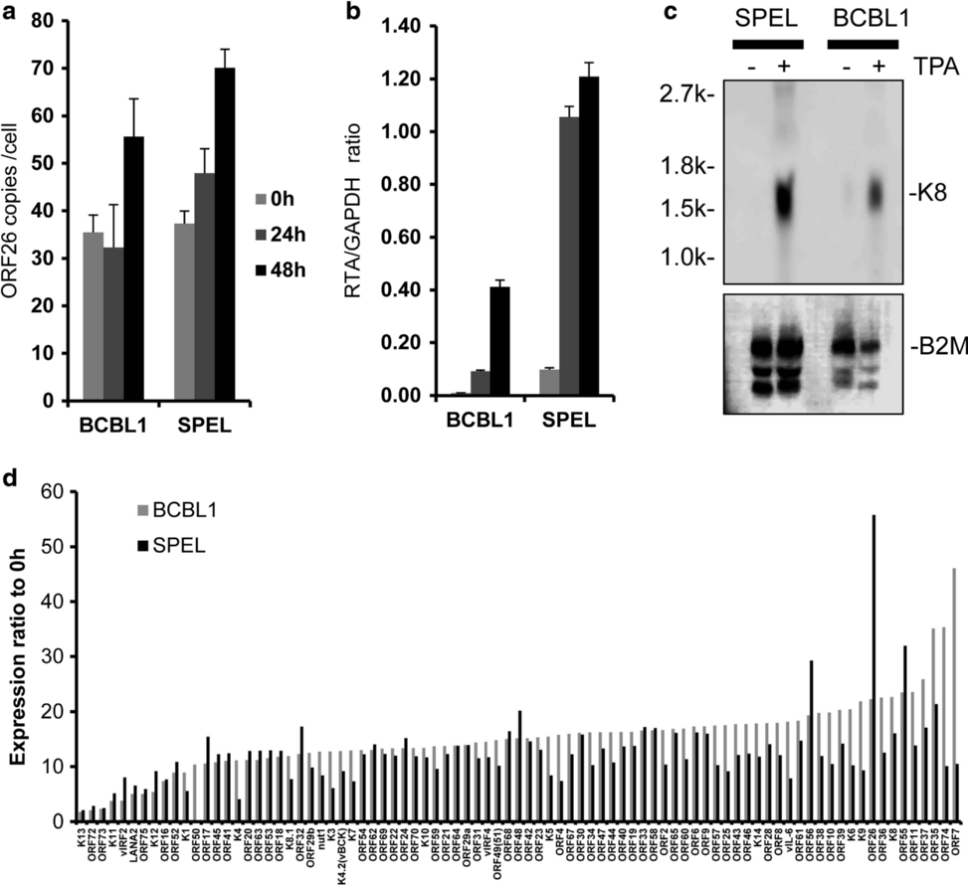
KSHV-encoded mRNA was induced by TPA in SPEL cells. **a** Real-time PCR analysis to determine the number of KSHV copies per cell in two TPA-stimulated cell lines. **b** Real-time RT-PCR analysis of the mRNA level of RTA in TPA-stimulated cells. **c** Northern blot analysis of KSHV-encoded transactivator protein, K8 (upper panel), and the human beta-2 microglobulin gene (B2M, lower panel). **d** KSHV real-time PCR array. The Y axis indicates the expression ratio to non-stimulated cells. KSHV-encoded mRNAs are listed in the graph in order of their expression levels in BCBL1 cells

**Fig. 8:**
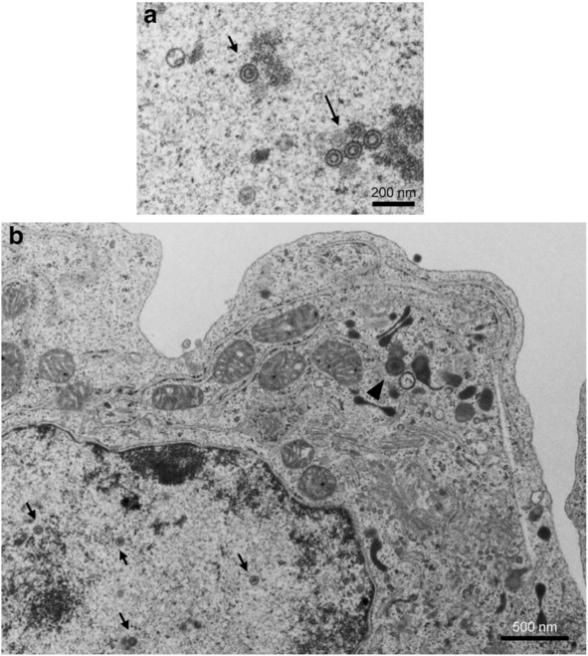
Transmission electron microscopy of KSHV particles in TPA-stimulated SPEL cells. **a** Virus capsids in the nucleus. Virus capsids are indicated by arrows. **b** Low power image of the cell. Virus capsids were observed in the nucleus (arrows) and arrow heads indicate enveloped virus particles in the cytoplasm. Bars indicate the scale

An immunofluorescence assay and western blot analysis demonstrated that TPA induced expression of RTA and other early lytic proteins encoded by KSHV, such as vIL-6, K8, and ORF59, in SPEL and BCBL-1 cells (Fig. 6). The localization of lytic proteins was consistent with the findings in other KSHV-positive cell lines [20]. The addition of TPA to the culture medium significantly increased viral DNA copy compared with non-treated cells (Fig. 7a). Real-time RT-PCR demonstrated an increase in the mRNA copy number of KSHV-encoded RTA in TPA-stimulated SPEL cells as well as in BCBL1 cells (Fig. 7b). Northern blot analysis showed that TPA induced the expression of K8 mRNA, an early gene of KSHV in SPEL cells (Fig. 7c). We also investigated the expression profiles of KSHV-encoded gene transcripts using a real time RT-PCR array that detected KSHV gene transcripts. The expression profiles of KSHV gene transcripts in SPEL cells were similar to those in BCBL-1 cells (Fig. 7d). Finally, electron microscopic analysis revealed an increased number of enveloped viral particles in the cytoplasm and viral capsid in the nucleus of TPA-stimulated SPEL cells (Fig. 8). These data indicated that KSHV lytic infection was induced by TPA stimulation in SPEL cells as well as in another KSHV-positive PEL cell line, BCBL1.

### KSHV genome sequence analysis in SPEL cells

**Fig. 9:**
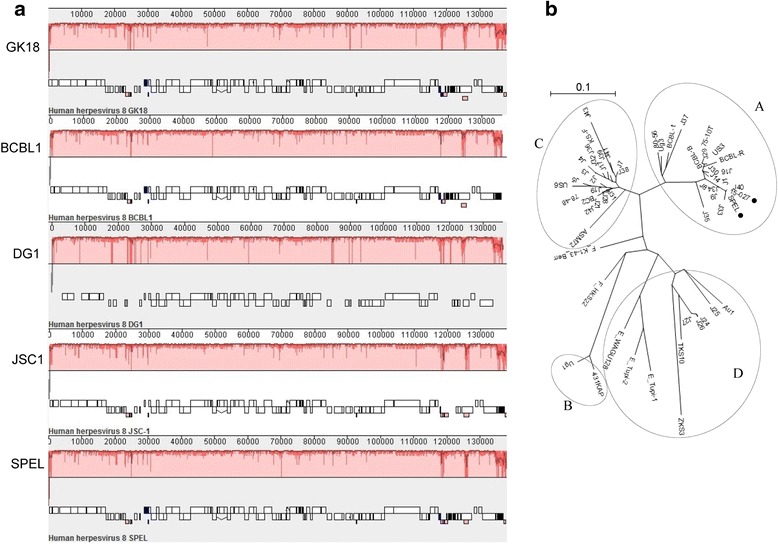
Full genome sequencing of KSHV from SPEL cells. **a** Distribution of nucleotide variations across the KSHV genome compared with the reference sequence GK18. Nucleotide similarity is indicated by red lines. Annotated open reading frames are shown as white rectangles below the similarity bars. This figure was generated using Mauve software. **b** Phylogenetic tree of the KSHV K1 region. SPEL cells and their origin cells (15-027) are indicated by small black circles, both belong to group A

KSHV particles were collected from the supernatant of stimulated SPEL cells, and the DNA of KSHV was subjected to deep sequencing using a next-generation sequencer. The complete genome of KSHV in SPEL cells was found to be 137,988 bp in length and contain terminal repeats. Compared with the reference sequence of KSHV (strain GK18), nucleotide variations were identified with other representative KSHV strains (Fig. 9a). Internal repeat regions in the DNA from SPEL cells (between K4.2 and K5, ORF69 and ORF71, and internal repeat in LANA-1) were unique compared with other strains. Two single nucleotide polymorphism (SNP) sites were identified in the exon of ORF56 (G/C nt 80190) and between ORF57 and vIRF1 (A/G nt 83625). The SNP in ORF56 leads to a non-synonymous mutation, with a change of a glycine or histidine in the 257 position of the protein. A phylogenetic tree based on the sequence of the K1 region showed that KSHV from SPEL cells belonged to genotype A, along with the original PEL cells of the patient and other AIDS-associated PEL and Kaposi’s sarcoma patients in Japan (Fig. 9b).

### Tumor formation in SCID mice by inoculation of SPEL cells

**Fig. 10:**
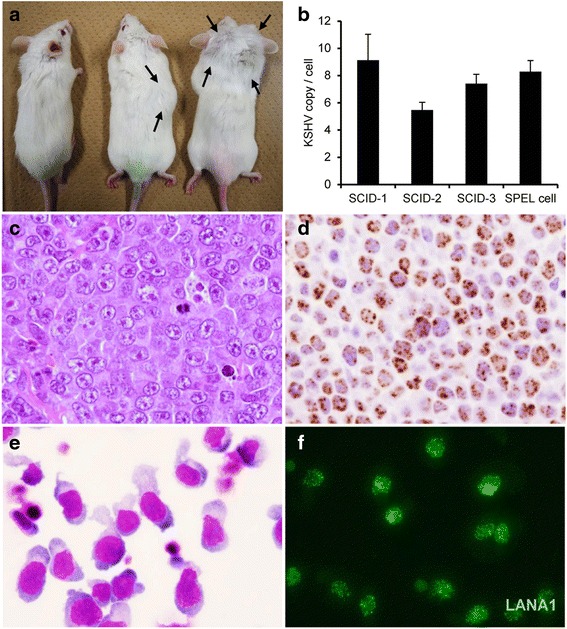
Tumor formation mediated by SPEL cells in vivo. **a** Macroscopic view of SPEL-inoculated SCID mice 4 weeks after inoculation. Mice in the middle and right displayed solid tumors at the inoculation sites (arrows). The mouse on the left was the control with no inoculation. **b** Analysis of the number of KSHV genome copies in the solid tumors of SCID mice and SPEL cells by real-time PCR. Error bars indicate the standard deviations. **c**, **d** HE staining (**c**) and immunohistochemistry for LANA-1 (**d**) of the resected tumors from SCID mice. **e**, **f** Giemsa staining (**e**) and immunofluorescence for LANA-1 of cultured cells from SCID tumors (**e**)

To investigate tumor formation in vivo, SPEL cells were inoculated into the posterior cervical region of three SCID mice. After 4–5 weeks, solid tumors 2–3 cm in diameter were observed at the inoculation site of each mouse (Fig. 10). Tumors also occurred in the back in two mice, and near the face in one mouse. Pathological analysis revealed that these tumors consisted of large diffuse lymphoma cells. LANA-1 was detected in the nucleus of almost all of the tumor cells by immunohistochemistry. Real-time PCR demonstrated that the KSHV and human β-actin DNA counts were similar to those in SPEL cells (Fig. 10b). These data provided evidence that all of these tumor cells were derived from SPEL cells. Cells derived from the resected tumors of SCID mice were cultured in RPMI1640 medium with 10 % FBS. The cells showed autonomous growth in the culture medium for at least four months, and were characterized as LANA-1-positive lymphoma cells with a gourd-shaped morphology, indicating that the cells originated from SPEL cells (Fig. 10e and f).

### HDAC inhibitors induced KSHV replication in SPEL cells

**Fig. 11:**
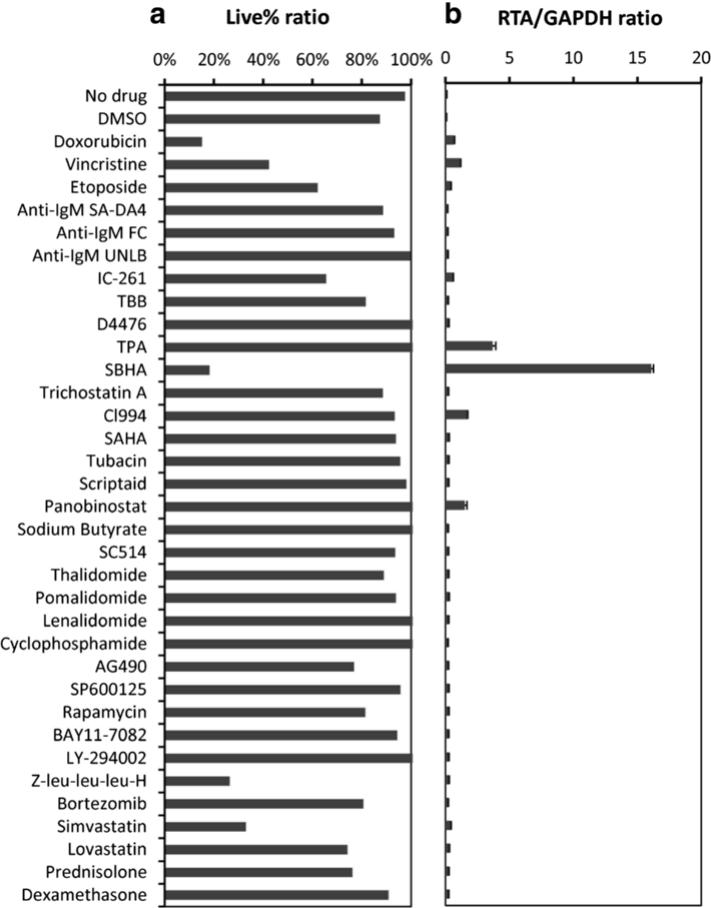
Drug screening for cell death and KSHV activation in SPEL cells. **a** Cell viability at 48 h after the addition of a drug to the culture medium. **b** Expression ratio of RTA to GAPDH mRNA at 48 h after the addition of a drug to the culture medium by real-time RT-PCR. Detailed information regarding the drugs and concentrations used are listed in Table 1

To test the effect of various drugs on SPEL cells, 34 drugs were individually added to SPEL cells and the cells were cultured for 48 h. A cell viability test demonstrated that SBHA, an HDAC inhibitor, induced cell death in SPEL cells in a similar way to other anti-cancer drugs such as doxorubicin and vincristine (Fig. 11a). SBHA also induced the expression of RTA transcripts in SPEL cells, as seen for other HDAC inhibitors such as tacedinaline (CI-994), and panobinostat (LBH589) (Fig. 11b and data not shown). The copy number of ORF26 was also increased in both cell lines stimulated with SBHA and panobinostat (data not shown). These data suggested that HDAC inhibitors may contribute to the killing of tumor cells by inducing KSHV lytic infection from a latent infection in these cells.

## Discussion

In the present study, we established a new KSHV-positive and EBV-negative PEL cell line, SPEL. To date, 10 KSHV-positive/EBV-negative PEL cell lines have been reported including SPEL (Table 2) [11–16]. The morphological features of SPEL cells, a gourd-shaped form with a polarized nucleus, were quite different from those of previous cell lines. This morphology was maintained after inoculation into SCID mice. Although the histological features of solid tumors in SCID mice showed diffuse large cell morphology similar to diffuse large B-cell lymphoma cells, culture cells established from resected tumors from the SPEL-inoculated SCID mice demonstrated a gourd-shaped form similar to SPEL cells. This suggested that the gourd-shaped form was associated with SPEL cell growth in effusion. The cytoplasm of SPEL cells contains abundant mitochondria and Golgi apparatus. In addition, the polarized localization of caveolin, an integral membrane protein associated with caveolae, and transferrin receptor were observed in the cytoplasm of SPEL cells, suggesting that such morphological features may be associated with protein production and signal transduction in these cells.

**Table 2:** KSHV^+^ EBV^˗^ PEL cell lines reported previously

No.	Name of cell line	Reference	Patient	Site	HIV infection	KSHV	EBV	Lymphoid marker	B cell marker			T cell marker		Activation marker		Plasma cell marker
								CD45	CD19	CD20	CD79a	CD3	CD4	CD30	CD38	CD138+
1	AP3	[36]	42 M	Ascites	yes	+	−	+	−	NT	−	−	NT	+	+	+
2	AP6	[36]	26 M	Pleural effusion	yes	+	−	NT	−	NT	−	−	NT	+	+	+
3	BC3	[37]	85 M	Pleural effusion	no	+	−	+	−	−	NT	−	−	+	+	NT
4	BCBL1	[15]	40 M	Ascites	yes	+	−	UN	UN	UN	UN	UN	UN	UN	UN	UN
5	BCP1-	[38]	94 M	Pleural effusion	no	+	−	−	−	−	−	−	NT	−	+	NT
6	HH-B2	[39]	UN	Pleural effusion	yes	+	−	+	−	−	NT	−	−	−	+	NT
7	TY1	[14]	47 M	Pericardial effusion	yes	+	−	+	−	−	UN	−	−	+	NT	NT
8	VG1-	[40]	59 M	Pleural effusion	no	+	−	−	−	−	−	−	NT	+	+	NT
9	GTO	[16]	39 M	Pericardial effusion	yes	+	−	+	−	−	−	−	+	−	+	+
10	SPEL	This study	49 M	Pleural effusion	yes	+	−	+	−	−	−	−	−	+	+	+

*NT* not tested. *UN* unknown

The complete genome of KSHV from SPEL cells was sequenced in this study. Although several full KSHV genomes have previously been deposited in the GenBank database, none of these originated from Asia. Phylogenetic tree analysis based on the K1 region revealed that SPEL KSHV belongs to genotype A. Nucleotide variations, including SNPs and different repeat numbers in the repeat region, were detected in SPEL KSHV compared with the genome sequence of another genotype A strain of KSHV, BrK.219 (accession no. KF588566). However, chemical stimulation with TPA was able to induce activation and replication of KSHV, suggesting that the nucleotide variations identified in SPEL KSHV did not affect viral activation.

It has been reported that several chemical and biological agents induce the viral lytic cycle in PEL cell lines [15, 22, 30], the best studies of which are TPA [15] and HDAC inhibitors [30]. HDACs are enzymes that have the ability to remove acetyl groups from ε-*N*-acetyl lysine amino acids in histones and other proteins. HDACs are critical regulators of both nuclear and cytoplasmic processes, including transcriptional initiation and elongation, protein stability, and multi-protein complex formation [31–34]. In recent years, HDACs have been intensively investigated as therapeutic targets because of their important role in gene expression. Some HDAC inhibitors such as sodium butylate, valproic acid, trichostatin A, nicotinamide, and sirtinol have been reported to induce viral replication in PEL cell lines [30, 35]. Although the mechanism of action of HDAC inhibitors in PEL cells have yet to be fully elucidated, HDAC inhibitors are thought to play multiple roles in expressing RTA of KSHV, which leads to viral DNA replication and the release of mature virions, resulting in tumor destruction [35]. In our experiments, SBHA strongly induced KSHV lytic replication in SPEL cells and decreased cell viability compared with other HDAC inhibitors. A limitation of our experiments was that we were unable to clarify whether the cytotoxicity in SPEL cells was due predominantly to viral lytic re-activation or the induction of apoptosis. Despite this, the establishment of the SPEL cell line may further our understanding of the pathogenesis of PEL and the therapeutic mechanism of action of HDAC inhibitors in KSHV-related malignancies.

## Conclusions

A new KSHV-positive and EBV-negative PEL cell line, SPEL was established. SPEL cells showed gourd-shaped morphology with a polarized nucleus. TPA and SBHA, a HDAC inhibitor, induced expression of KSHV lytic proteins and the production of KSHV particles in SPEL cells. Next-generation sequencing revealed the 138 kbp genome sequence of KSHV in SPEL cells. This cell line may contribute to furthering our understanding of the pathogenesis of PEL and KSHV infection.

## Abbreviations

EBV, Epstein-Barr virus; HDAC, histone deacetylase; KSHV, Kaposi’s sarcoma-associated herpesvirus; LANA, latency-associated nuclear antigen; PEL, primary effusion lymphoma; RTA, replication and transcription activator; RT-PCR, reverse transcription polymerase chain reaction; SBHA, suberic bishydroxamate; SCID, severe combined immunodeficiency; TPA, tetradecanoylphorbol acetate
